# Tumor-targeted delivery of lnc antisense RNA against RCAS1 by live-attenuated tryptophan-auxotrophic *Salmonella* inhibited 4T1 breast tumors and metastasis in mice

**DOI:** 10.1016/j.omtn.2023.102053

**Published:** 2023-10-13

**Authors:** Chandran Sivasankar, Chamith Hewawaduge, Pandiyan Muthuramalingam, John Hwa Lee

**Affiliations:** 1 College of Veterinary Medicine, Jeonbuk National University, Iksan Campus 54596, Republic of Korea; 2 Department of GreenBio Science, Gyeongsang National University, Jinju 52725, South Korea

**Keywords:** MT: delivery strategies, RCAS1, antisense-RNA, tryptophan-auxotroph, *Salmonella* delivery, tumor specificity, 4T1 breast cancer, anti-tumor effect

## Abstract

Emerging chemo- and radiotherapy resistance exacerbated the cancer risk and necessitated novel treatment strategies. Although RNA therapeutics against pro-oncogenic genes are highly effective, tumor-specific delivery remains a barrier to the implementation of this valuable tool. In this study, we report a tryptophan-auxotrophic *Salmonella* typhimurium strain as an onco-therapeutic delivery system with tumor-targeting ability using 4T1 mice breast-cancer model. The receptor-binding cancer antigen expressed on SiSo cell (RCAS1) is a cancer-specific protein that induces the apoptosis of peripheral lymphocytes and confers tumor immune evasion. We designed a long non-coding antisense-RNA against RCAS1 (asRCAS1) and delivered by *Salmonella* using a non-antibiotic, auxotrophic-selective, eukaryotic expression plasmid, pJHL204. After *in vivo* tumor-to-tumor passaging, the JOL2888 (Δ*trpA*, Δ*trpE*, Δ*asd* + asRCAS1) strain exhibited high sustainability in tumors, but did not last in healthy organs, thereby demonstrating tumor specificity and safety. RCAS1 inhibition in the tumor was confirmed by western blotting and qPCR. In mice, JOL2888 treatment reduced tumor-associated macrophages, improved the T cell population, elicited cell-mediated immunity, and suppressed cancer-promoting genes. Consequently, the JOL2888 treatment significantly decreased the tumor volume by 80%, decreased splenomegaly by 30%, and completely arrested lung metastasis. These findings highlight the intrinsic tumor-targeting ability of tryptophan-auxotrophic *Salmonella* for delivering onco-therapeutic macromolecules.

## Graphical abstract

**Figure:**
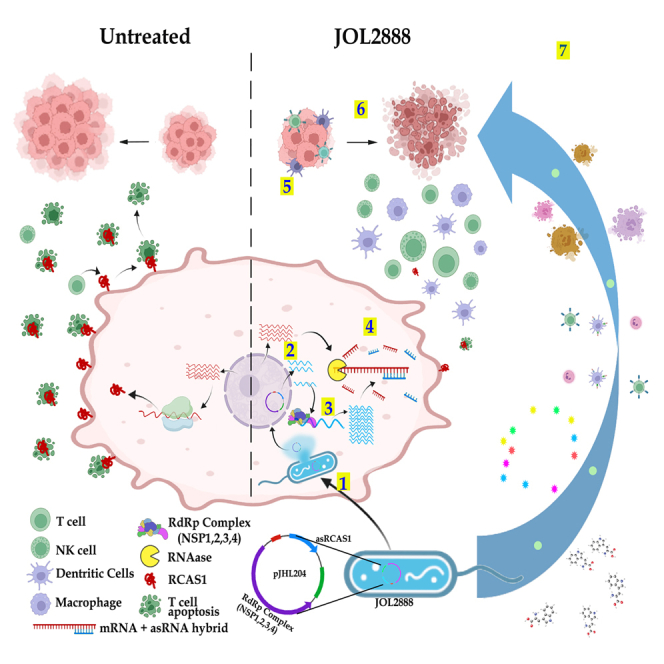

## Teaser

Lee and colleagues demonstrated the anti-tumor potential of tumor-specific tryptophan auxotrophic *Salmonella*, that delivers lnc asRCAS1. The anti-tumor effect was determined by improved T cell population, reduced TAMs levels, suppression of pro-oncogenic genes, and immune cell infiltration in the tumor.

## Introduction

Cancer continues to be one of the top diseases causing human mortality (https://www.cdc.gov/nchs/fastats/leading-causes-of-death.htm), with an incidence rate exceeding 18 million cases per year globally (https://www.wcrf.org/cancer-trends/worldwide-cancer-data/). The recurrence of malignancies that are resistant to chemo- and radiotherapy is the major reason for treatment failure.^1^ Therefore, existing treatments need more advancement, including combinations with novel strategies. The receptor-binding cancer antigen expressed on SiSo cells (RCAS1) is a cancer-specific protein that strongly correlates with cancer growth.^2^ It is also known as estrogen receptor-binding fragment-associated gene 9 (EBAG9). RCAS1 induces peripheral lymphocytes to undergo apoptotic cell death, thereby conferring immune evasion to tumor cells.^2^ Additionally, RCAS1 expression has been implicated in tumor invasiveness.^3^ It has been shown that the expression of RCAS1 is associated with the development of immunosuppressive macrophages.^4^ An examination of human cancer tissue samples showed that 40%–65% of patients had cancer cells and macrophages that were RCAS1 immuno-positive.^5^ RCAS1 also plays a major role in tumor stromal remodeling.^6^ Previous *in vitro* studies showed that inhibiting RCAS1 expression improved the T cell population, and thereby quenched immune evasion.^7^^,^^8^

Long non-coding RNAs (lncRNAs), which range from 200 bp to kilobases long, are involved in a variety of healthy cellular functions and disease conditions.^9^ Highly specific gene silencing is achieved by lnc antisense RNA (asRNA) due to its large size.^9^ Naturally, cancer cells express cis-/trans-acting lnc asRNAs to suppress apoptosis-related genes and enhance their viability.^10^ The same strategy can be used against cancer to inhibit pro-oncogenic genes and decrease tumor progression.^11^ RNA therapies are an emerging paradigm to combat various cancers and are being studied at various levels. Although RNA silencing can be used therapeutically *in vivo*, intracellular delivery across the plasma membrane and intratumoral delivery are challenging.^12^ To address the issues of plasma membrane transport, intracellular delivery, sensitivity to RNAase, and endosomal escape, small interfering RNA (siRNA), short hairpin RNA, microRNA, asRNA, and lncRNA have been delivered using viral vectors, nanoparticles, lipid nanoparticles, and so on.^12^ However, most of these methods still exhibit only moderate anti-tumor effects because the therapeutic RNAs are either delivered to undesirable sites or do not reach the tumors in adequate amounts because of degradation by nucleases.^12^ Additionally, these systems cannot kill latent tumor cells that are away from the vasculature or cells in metastatic tumors because of the distinct microenvironments. Moreover, viral delivery systems are expensive to implement.^13^^,^^14^ The development of safe, affordable, and effective *in vivo* systems to deliver inhibitory RNAs into tumor cells is critical to their success in treating cancer. The ideal delivery vehicle would be able to deliver the therapeutic agent effectively and specifically to the tumor while being nontoxic to normal cells.

In recent years, bacteria-mediated cancer treatment against drug and radiation resistant cancers has gained increasing attention. *Salmonella* typhimurium (ST) is one of the top bacteria studied for onco-therapeutics. It has the advantage of causing widespread, systemic infection, making it applicable against any type of tumor.^15^ As a facultative anaerobe, it can penetrate and proliferate in both vascularized and hypoxic regions of tumors.^16^^,^^17^ *Salmonella* is also a well established delivery vehicle for antigens, DNA, or RNA for immunogenic and therapeutic prospects.^18^^,^^19^ The engineering plasticity of the *Salmonella* genome facilitates the development of safe and non-virulent cancer-specific auxotrophic strains.^20^^,^^21^

In this study, we used ST for the tumor-targeted delivery of asRNA against RCAS1, evaluated the asRCAS1 suppression of RCAS1 in the tumor, and assessed the effect of the treatment on tumor growth, metastasis, tumor-associated macrophages, pro-oncogenic gene expression, and the T cell population level in tumor-bearing mice. In our previous study, we developed a tryptophan auxotrophic ST strain (ST2514-Δ*trpA*, Δ*trpE*) that can stringently grow in a tryptophan-abundant niche.^22^ The strain exhibited high tumor specificity; therefore, we modified our previously reported cancer-targeting *Salmonella* strain into a delivery vehicle for asRCAS1 by further deleting the *asd* gene (aspartate-semialdehyde dehydrogenase), which is complemented by a delivery plasmid pJHL204. The pJHL204 is a eukaryotic expression system with a Semliki Forest virus (SFV) replicon, consisting of non-structural proteins (NSPs) 1–4 that form an RNA-dependent RNA polymerase (RdRp) complex. The system independently enhances asRNA expression in the cytoplasm, which can lead to improved outcomes.^23^ In this study, the *Salmonella*-delivered lncRNA asRCAS1 inhibited RCAS1, tumor growth, and metastasis in murine breast cancer. In addition, we evaluated the tumor specificity and safety of the therapeutic strain. Overall, this proof-of-concept study demonstrates that tryptophan auxotrophic *Salmonella* can deliver asRCAS1 emphasizing its triple advantages of onco-specific deliverability, therapeutic effectiveness, and the suppression of immune evasion via T cell immune activation.

## Results

### *In vitro* anti-cancer effects

**Figure 1:**
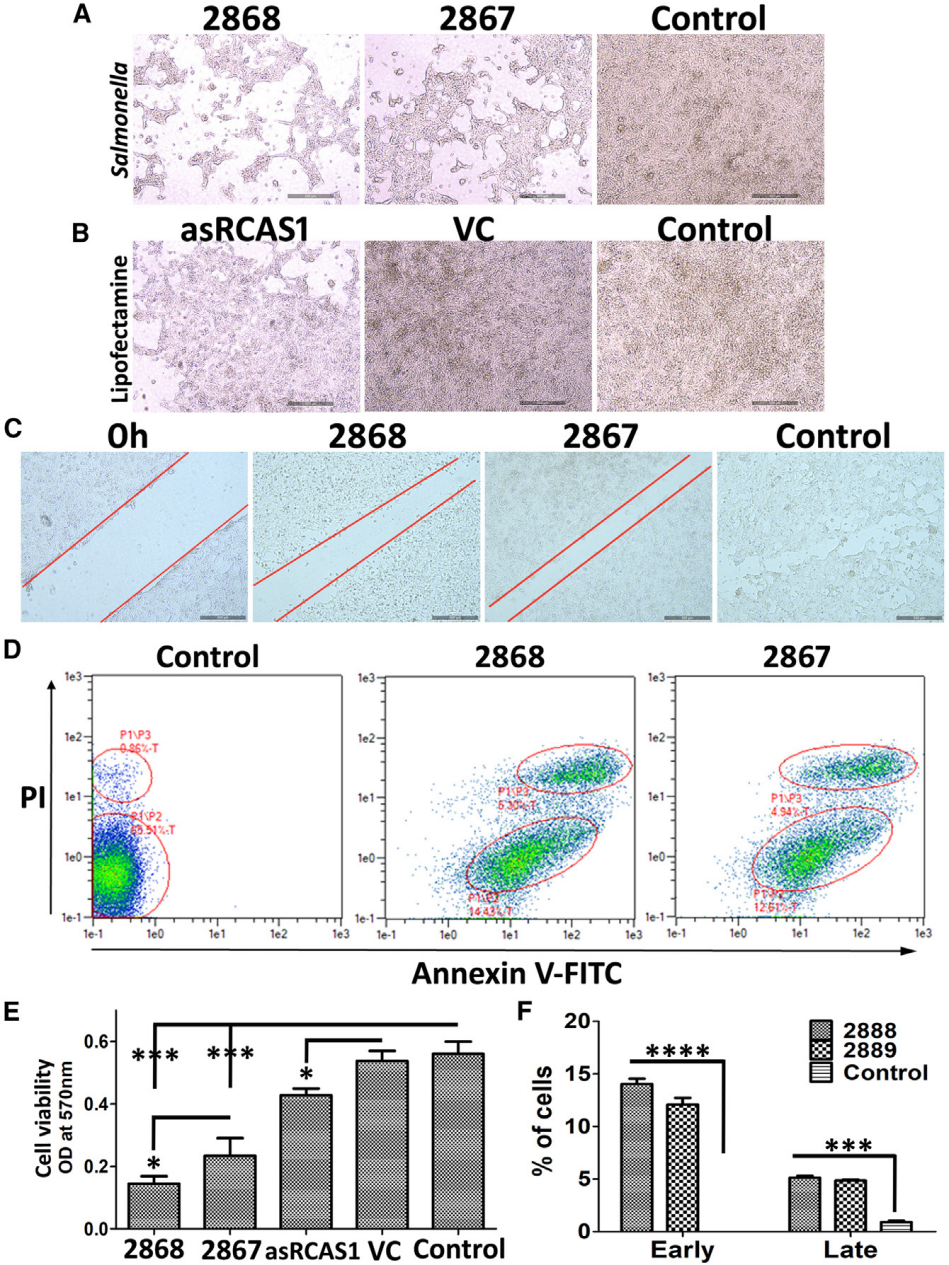
Effects of *Salmonella-* and lipofectamine-mediated delivery of asRCAS1 cloned plasmid on 4T1 cell proliferation *in vitro* (A) Representative microscopic images of cells subjected to asRCAS1-*Salmonella* bactofection and (B) asRCAS1 lipofectamine-transfection. The VC indicates the vector control, i.e., only plasmid without asRCAS1. (C) Representative micrographs of wound healing assay showing the reduction of cancer cell proliferation by asRCAS1-delivering *Salmonella* (JOL2868). (D) Representative images of apoptosis FACS analysis dot plot (E). Bar diagram of MTT assay, optical density (OD) at 570 nm indicating the number of viable cells. (E) Annexin V- PI FACS plot showing the induction of apoptosis by JOL2868 in 4T1 mouse breast cancer cells. The experiment was performed thrice with three biological replicates each. The data were analyzed by ANOVA using Tukey’s post hoc test. ∗p < 0.05; ∗∗p < 0.01; ∗∗∗p < 0.001.

The 4T1 cancer cells were transfected with the asRCAS1 plasmid construct and asRCAS1-delivering *Salmonella* JOL2868 (Δ*trpA ΔtrpE Δasd* + pJHL270 + lnc-asRCAS1). The MTT assay and microscopic visualization revealed a significant decrease in cell proliferation, 58%–74%, after *Salmonella*-based bactofection, and JOL2868 was significantly better that vector control strain JOL2867. In contrast, lipofectamine transfection of the asRCAS1 plasmid construct produced only mild inhibition, of about 20%, and no considerable difference was noted in vector control (Figures 1A–1C and 1E). Based on the cell proliferation results, the wound healing assay was done by *Salmonella* bactofection using JOL2868 and JOL2867. A substantial decrease in wound closure was observed with both treatments; however, the inhibition was considerably better with the JOL2868 carrying asRCAS1 than with the JOL2867 vector control strain (*ΔtrpA ΔtrpE Δasd* + pJHL270) (Figure 1C).

The anticancer effect of JOL2868 was further validated by annexin V-PI fluorescence-activated cell sorting (FACS) analysis to determine the induction of apoptosis. That result showed that the *Salmonella* treatment triggered both early and late apoptosis, as evidenced by annexin V+ and PI+, respectively. The apoptosis results indicated that both JOL2868 carrying asRCAS1 and vector control strain JOL2867 showed significant apoptosis induction, but there was no significant difference between them (Figures 1D and 1F).

### Safety and *in vivo* passage of therapeutic *Salmonella*

**Figure 2:**
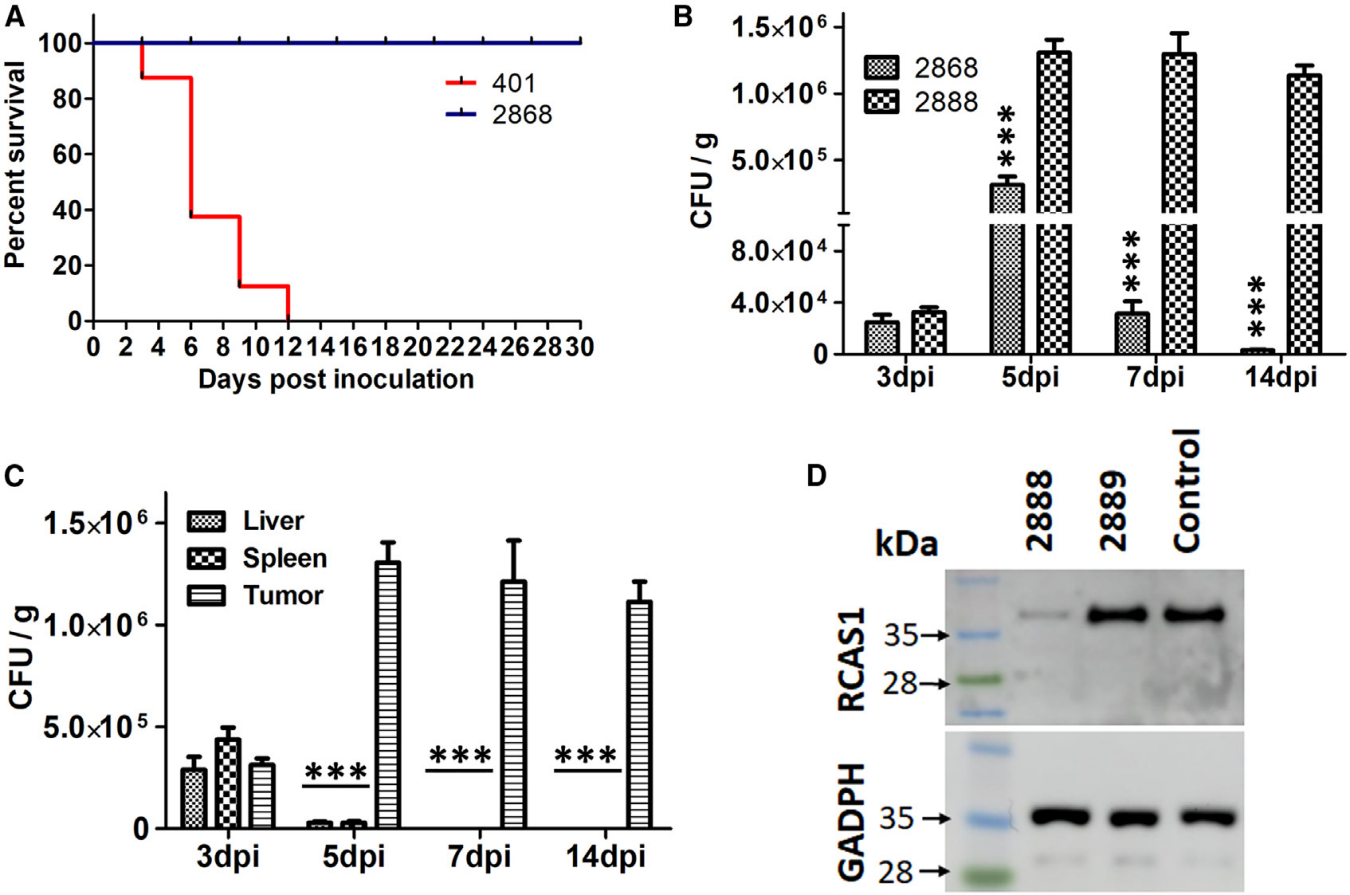
Safety and tumor specificity of asRCAS1-delivering *Salmonella* (A) The survival graph shows that JOL2868 produced no mortality in mice (n = 6). The experiment was performed twice. (B) CFU of *Salmonella* colonized in tumors at different time points. The tumor-to-tumor passaged strain (JOL2888) showed better tumor colonizing ability than the unpassaged strain (n = 2). The experiment was performed twice with three experimental replicates. The data were analyzed by Student’s t-test. ∗∗∗p < 0.001. (C) Bacterial load of JOL2888 in tumors and healthy organs at different time points. JOL2888 was sustainable only in tumors, not in healthy organs. The data were analyzed by ANOVA using Tukey’s post hoc test. ∗∗∗p < 0.001. (D) Western blot image of RCAS1 expression in tumors treated by the asRCAS1-delivering JOL2888 strain, the vector control JOL2889 strain, and the untreated control. The expression of GADPH was analyzed as the housekeeping control.

The safety of JOL2868 (carrying asRCAS1) was evaluated in mice after intraperitoneal systemic infection. JOL2868 infection did not noticeably alter the body weight (data not shown). There were no significant changes in food intake, body weight, or behavior after infection, and neither illness symptoms nor deaths were recorded. In the group infected with the JOL401 wild-type pathogenic strain, severe disease symptoms, such as ruffled fur, reluctant behavior, and curved body posture, were observed beginning the day after infection. Mice were started dying 3 days post infection (dpi), and all of them died within 12 dpi (Figure 2A).

To acquire high tumor specificity, *in vivo* passaging was done in tumor-bearing mice. After three *in vivo* passages, the therapeutic strain was designated as JOL2888 (passaged strain carrying asRCAS1). Then, the tumor colonization of the *in vivo* passaged JOL2888 and unpassaged JOL2868 strains was compared in tumor-bearing mice. The tumors were dissected from the *Salmonella*-treated mice from 3 to 14 dpi, and equal amounts of tumor tissues were subjected to a colony-forming unit (CFU) assay. The results revealed that the *in vivo* passaged strain colonized the tumor better than the unpassaged one. At 7 and 14 dpi, the bacterial load of the unpassaged strain was meager, not even 3 × 10^3^, whereas the passaged strain was significantly higher, 1 × 10^6^ (Figure 2B). Alongside, the CFU of the passaged strain JOL2888 (carrying asRCAS1) was determined in tumor, spleen and liver. The bacterial load was similar in all of the tissues at 3 dpi, but at the later time points, the passaged auxotrophic strain was found only in the tumor at approximately 1 × 10^6^, and not found in the normal organs (Figure 2C). Overall, the results indicate that the passaged auxotrophic strain was adapted to the tumor environment, but not to healthy organs.

To further confirm the safety of ST2888 (*in vivo* passaged carrying asRCAS1) body temperature and complete blood count (CBC) analysis were performed. After JOL401 wild-type infection, significant hypothermia was observed with a nearly 1.5°C decrease in surface body temperature. The reticulocytes, neutrophils, and monocytes levels were significantly increased and lymphocytes and platelets levels were reduced in the JOL401-infected mice, whereas in the ST2888 mild alterations or no significant differences were observed. These alterations in body temperature and blood cell components in wild-type infection imply the virulent systemic bacterial infection and safety value of ST2888 (Table S3).

### Inhibition of RCAS1 expression in the tumors

The passaged therapeutic *Salmonella* JOL2888 (*in vivo* tumor passaged JOL2868 (Δ*trpA ΔtrpE Δasd* + pJHL270 + lnc-asRCAS1) was used to treat cancer via intraperitoneal administration. After four doses of the treatment course, the tumors were excised from the animals, and tumor lysates were tested for RCAS1 expression through western blotting analysis. A densitometry analysis of western blot in ImageJ software showed that the RCAS1 level was drastically reduced, by 17-fold, in the JOL2888-treated tumors, whereas no considerable decrease was detected in the vector control group, even though JOL2889 (*in vivo* tumor passaged JOL2867 vector control strain [Δ*trpA ΔtrpE Δasd* + pJHL270]) exhibited moderate tumor reduction. The expression pattern was compared to GADPH as a housekeeping control, which showed similar expression in all tested samples (Figure 2D).

### Effect of JOL2888 treatment on the tumor-associated macrophage population

**Figure 3:**
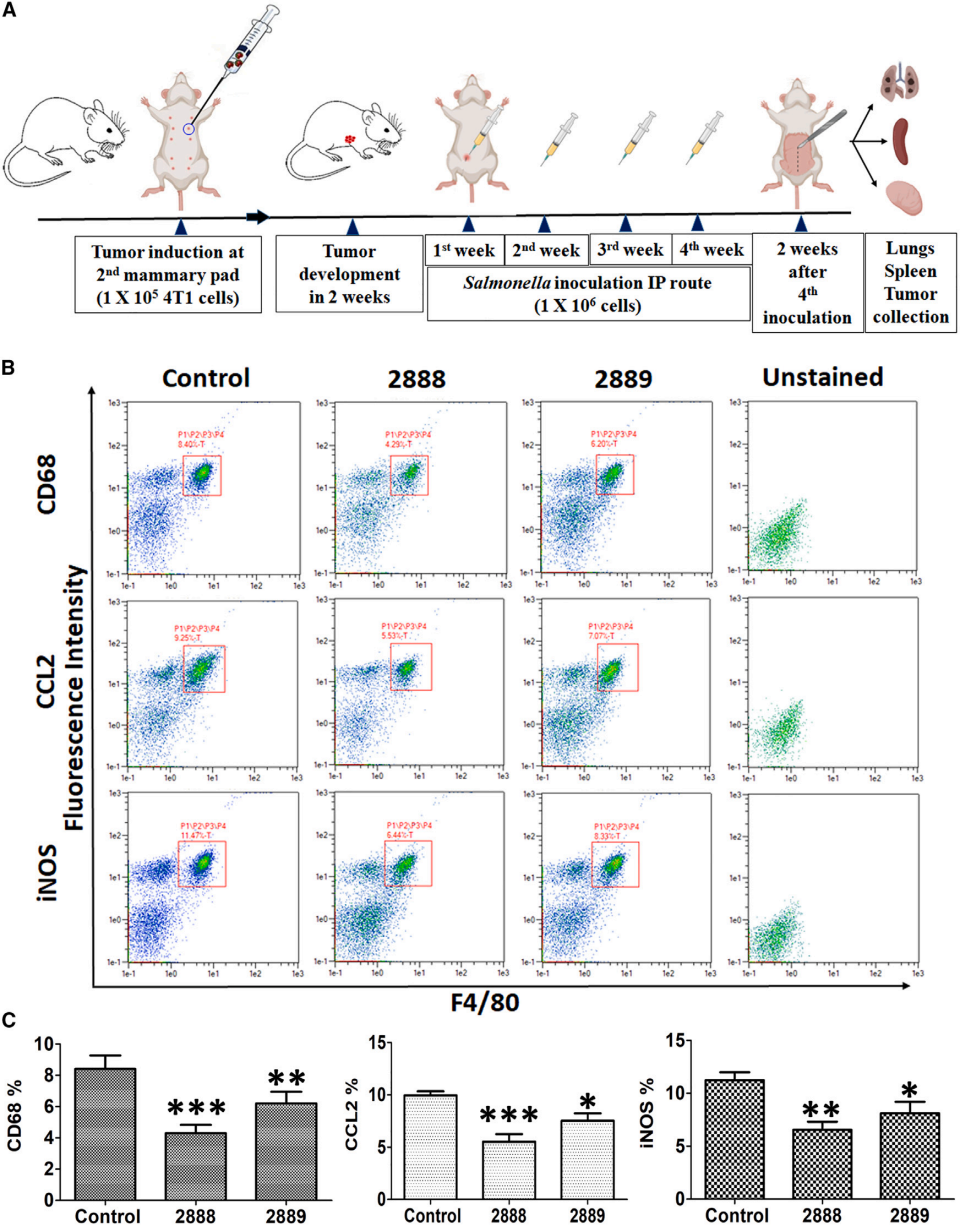
Effect of asRCAS1 *Salmonella* treatment on TAMs (A) Schematic diagram of tumor induction, *Salmonella* treatment, and analysis. (B) FACS plot depicting the levels of CD68, CCL2, and iNOS TAMs in control and treated tumors (n = 4). (C) Bar diagrams of TAM percentages in control and treated tumors. The experiment was performed twice with three experimental replicates. The data were analyzed by ANOVA using Tukey’s post hoc test. ∗p < 0.05; ∗∗p < 0.01; ∗∗∗p < 0.001.

The inhibition of the tumor-associated macrophage (TAM) population is indispensable to an anti-tumor effect.^24^^,^^25^ Therefore, three major TAMs, CD68, CCL2, and inducible nitric oxide synthase (iNOS), were tested. FACS analysis indicated a significant reduction of TAMs in the *Salmonella*-treated tumors. The JOL2888 that delivered asRCAS1 exhibited 33%–49% TAM reduction compared with the control, and the vector control auxotrophic *Salmonella* (JOL2889) exhibited 25%–34% reduction. Overall, the JOL2888 exhibited the better TAMs reduction activity (Figures 3B and 3C).

### Effect of JOL2888 treatment on the T cell population

**Figure 4:**
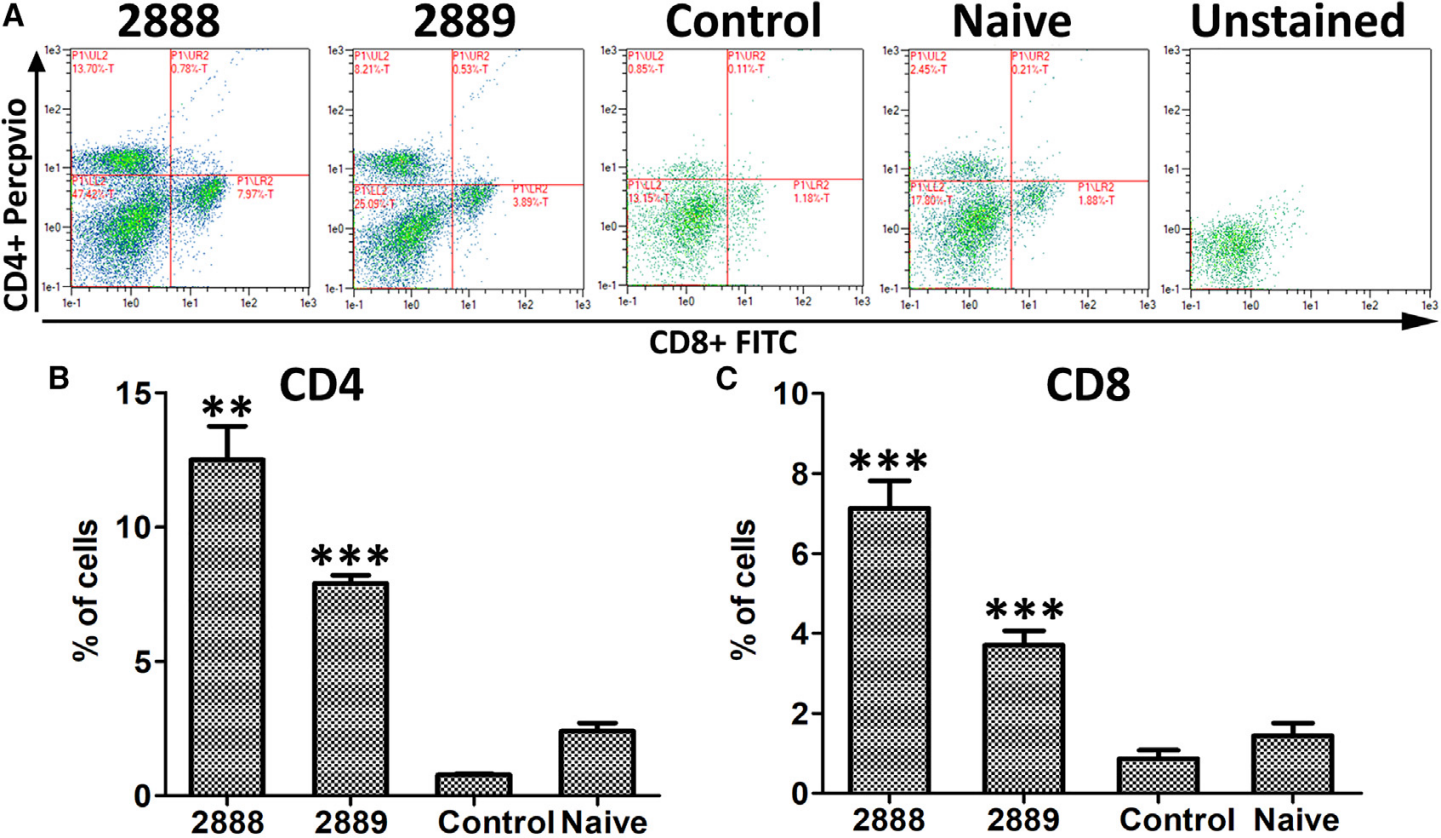
Effect of asRCAS1 *Salmonella* treatment on T cells (A) FACS plot depicting the levels of CD4^+^ and CD8^+^ T cells in control and treated mice. (B and C) Bar diagrams of CD4^+^ and CD8^+^ T cell percentages in control and treated mice (n = 4). The experiment was performed twice with three experimental replicates. The data were analyzed by ANOVA using Tukey’s post hoc test. ∗∗p < 0.01; ∗∗∗p < 0.001.

The RCAS1 produced by cancer cells is a known factor in immune evasion that induces apoptosis in lymphocytes, especially T cells. Therefore, the level of T cells was examined by FACS analysis. Splenocytes isolated from control and treated mice were subjected to FACS analysis after stimulation with 4T1 cell lyase as a recall antigen. We found that the untreated tumor-bearing mice had a very low T cell population, 3-fold less than healthy naive mice, whereas the JOL2888 treatment elicited a T cell response, with 13% and 8% of CD4^+^ and CD8^+^ cells, respectively. The naive mice were not exposed to any foreign antigens, either from *Salmonella* or cancer cells, and therefore showed only a basal level of CD4^+^ and CD8^+^ cells. Although T cell induction was seen with both *Salmonella* treatments, JOL2888 demonstrated a more pronounced improvement of 37% and 48% of CD4 and CD8 T cells, respectively, compared with vector control, caused by RCAS1 suppression (Figure 4).

### Effect of JOL2888 treatment on cancer- and RCAS1-associated gene expression

**Figure 5:**
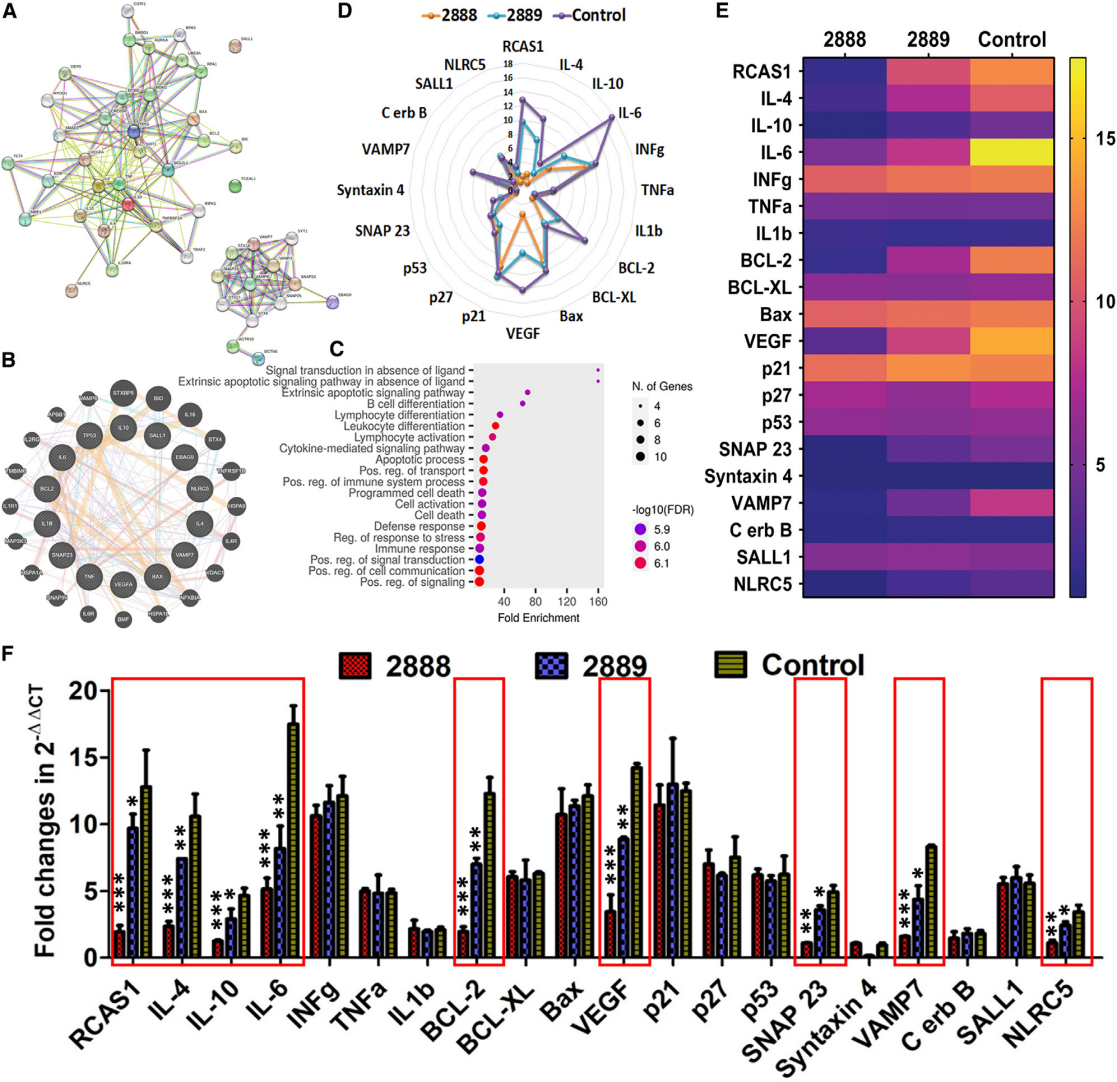
Gene network analyses of pro-oncogenic and RCAS1-linked genes and qPCR gene expression analysis (A) STRING analysis to determine gene interconnections, gene co-occurrence, gene co-expression, and gene neighborhood. (B) GeneMANIA analysis done to predict the genetic interactions and co-expression of selected genes. (C) Gene Ontology (GO) enrichments analysis of cancer-associated genes with their biological functions. The number of cancer-associated genes falling into each GO biological process is directly proportional to the ball size, and the balls are colored according to their significant enrichment value. The results in (A–C) illustrate the possible dysregulation of interconnected genes if the expression of any gene(s) is affected. A (D) radar chart, (E) heatmap, and (F) bar diagram of log_2_ fold changes in cancer-related gene expression between the control and *Salmonella*-treated samples. The experiment was performed thrice with three biological replicates each. The data were analyzed by ANOVA using Tukey’s post hoc test. ∗p < 0.05; ∗∗p < 0.01; ∗∗∗p < 0.001.

Reports on the connections between RCAS1 and other pro-oncogenic genes are scant; therefore, we set out to identify RCAS1-related proteins. Moreover, based on the previous literature,^26^^,^^27^ we selected important cancer-related genes, cell-cycle regulatory genes, and cytokine genes and analyzed their interconnections using *in silico* network analyses: STRING, GeneMANIA, and Kyoto Encyclopedia of Genes and Genomes (KEGG) enrichment.^28^^,^^29^ The network analyses indicated that most of the selected genes are highly interlinked and involved in regulating apoptosis, lymphocyte differentiation, cytokine-mediated signaling pathways, and stress responses. Moreover, the analyses also identified genes connected with RCAS1 (Figures 5A–5C). Of these genes, we selected 20 cancer-related genes, including RCAS1, and analyzed their expression levels in the tumors using qPCR. The results suggest that RCAS1 expression was drastically decrease in the JOL2888-treated tumors. In addition, cytokines such as IL-4, IL-10, and IL-6; the anti-apoptosis gene B cell lymphoma-2 (BCL-2); the angiogenesis gene vascular endothelial growth factor (VEGF); and the cancer proliferation and cell migration supporting genes synaptosome-associated protein 23 (SNAP23), vesicle-associated membrane protein 7 (VAMP7), and NLR family caspase recruitment domain-containing 5 (NLRC5) were found to be downregulated (Figures 5D–5F). Overall, of the 20 tested genes, 9 cancer-related genes were suppressed at a range of 2- to 12-fold, which implies that the asRCAS1-delivering JOL2888 restricted cancer cell proliferation, migration, vascularization, and metastasis (Figures 5D–5F).

### Arrest of the primary tumor and metastasis by JOL2888

**Figure 6:**
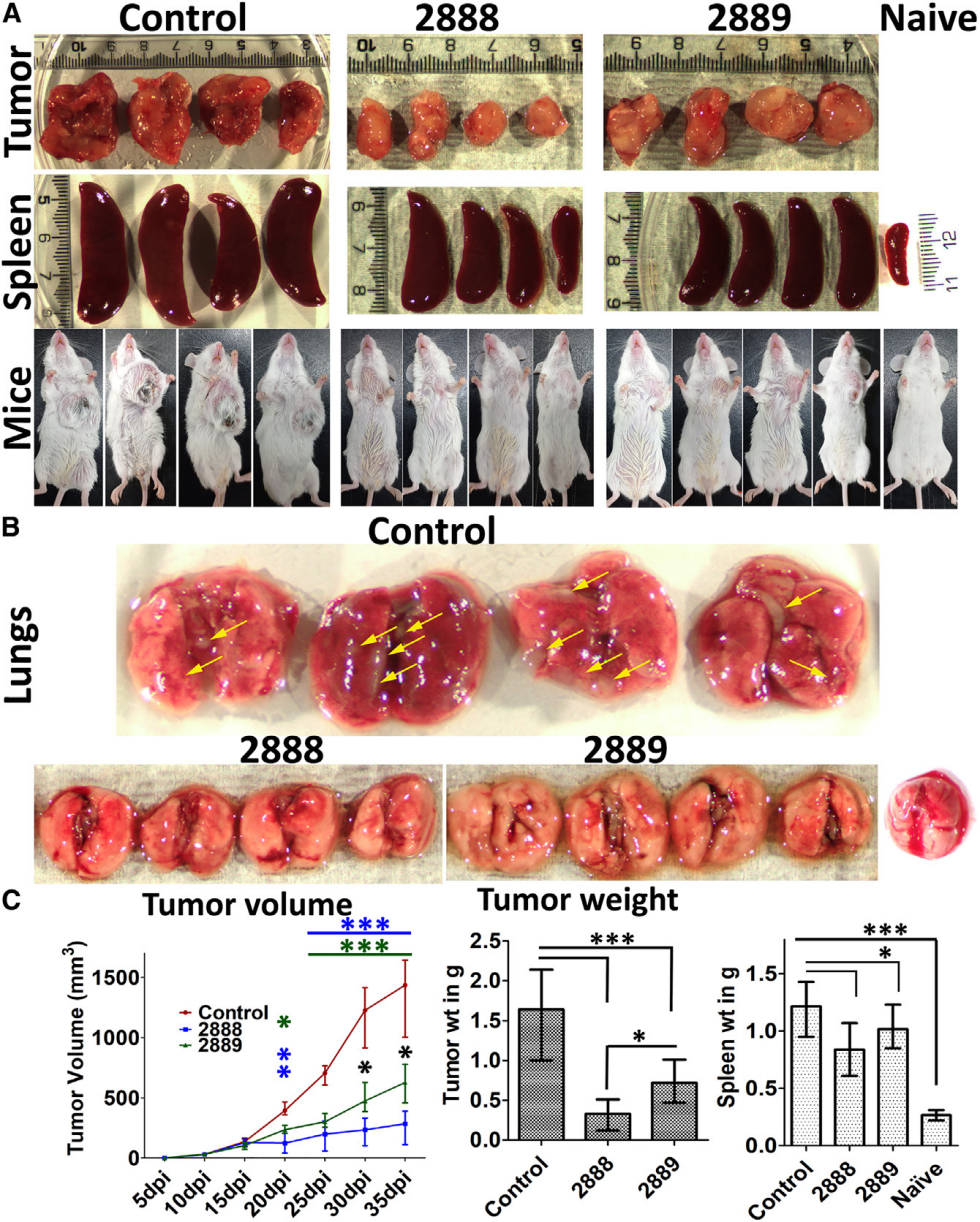
Evaluation of the therapeutic potential of asRCAS1-delivering *Salmonella* JOL2888 (A) Representative images of control, vector control, and treated tumors, spleens, and afflicted mice. (B) Representative images of lungs from control mice showing lung metastasis and *Salmonella*-treated lungs showing no lung metastasis (yellow arrows indicate metastatic nodules). (C) Line diagram of the tumor volume and Bar diagram of tumor and spleen weight. The experiment was performed twice (n = 4). The data were analyzed by ANOVA using Tukey’s post hoc test. ∗p < 0.05; ∗∗p < 0.01; ∗∗∗p < 0.001.

Tumor-bearing mice were treated with four doses of the *in vivo* passaged asRCAS1-delivering auxotrophic *Salmonella* (JOL2888) or the vector control (JOL2889). Then, all the mice were sacrificed, and their tumors, lungs, and spleens were collected and examined. Tumor size and volume were significantly reduced by approximately 56% and 80% by the JOL2889 and JOL2888 treatments, respectively, compared with the untreated control (Figures 6A and 6C). All the tumor-bearing mice were afflicted with cancer-induced splenomegaly; however, in the mice treated with JOL2888, the degree of splenomegaly was 30% lower than in the control group, although this was not a significant decrease compared with the vector control (Figures 6A and 6C). These findings show that JOL2888 (asRCAS1 delivering) outperformed JOL2889 (vector control) in preventing the growth of primary tumors because of its inhibition of RCAS1 by asRCAS1. Gross examination of the lungs showed that neither of the *Salmonella* treatment groups showed any evidence of metastatic malignancy, whereas the untreated control group showed many metastatic nodules (Figure 6B).

### Histopathological analysis

**Figure 7:**
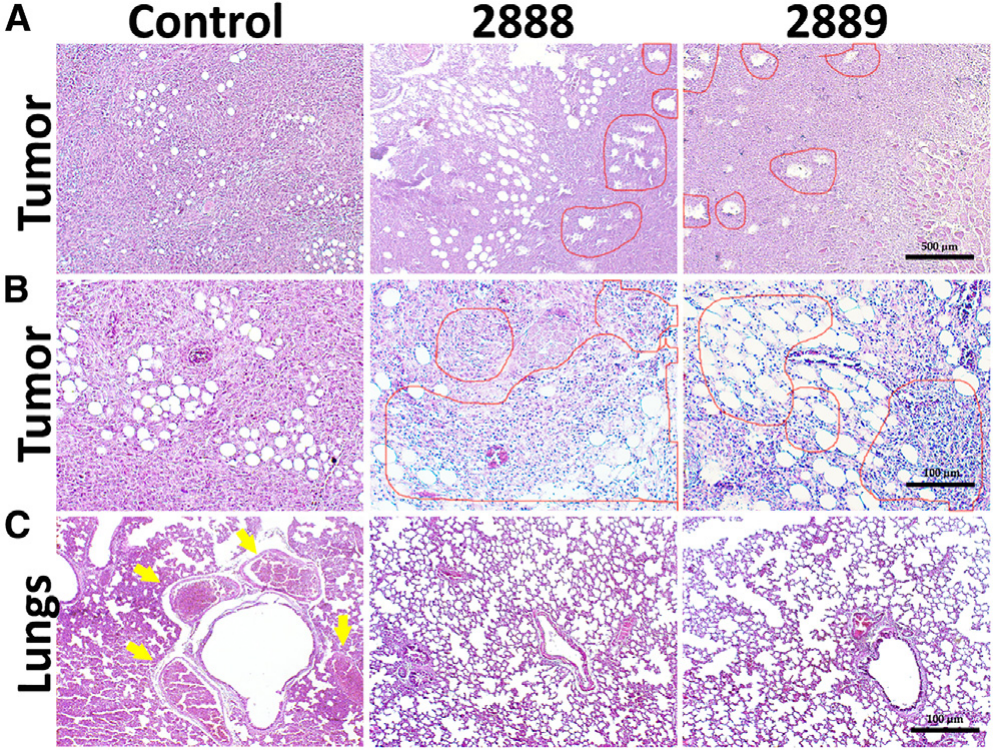
Microscopic images of H&E-stained tumor and lung samples (A) Tumor samples at 100× magnification; red circles indicate the necrotic foci caused by *Salmonella* infection. (B) Tumor samples at 200× magnification; red circles indicate the infiltrated immune cells after *Salmonella* infection. (C) Tumor samples at 200× magnification; yellow arrows indicate micro-metastatic nodules.

**Figure 8:**
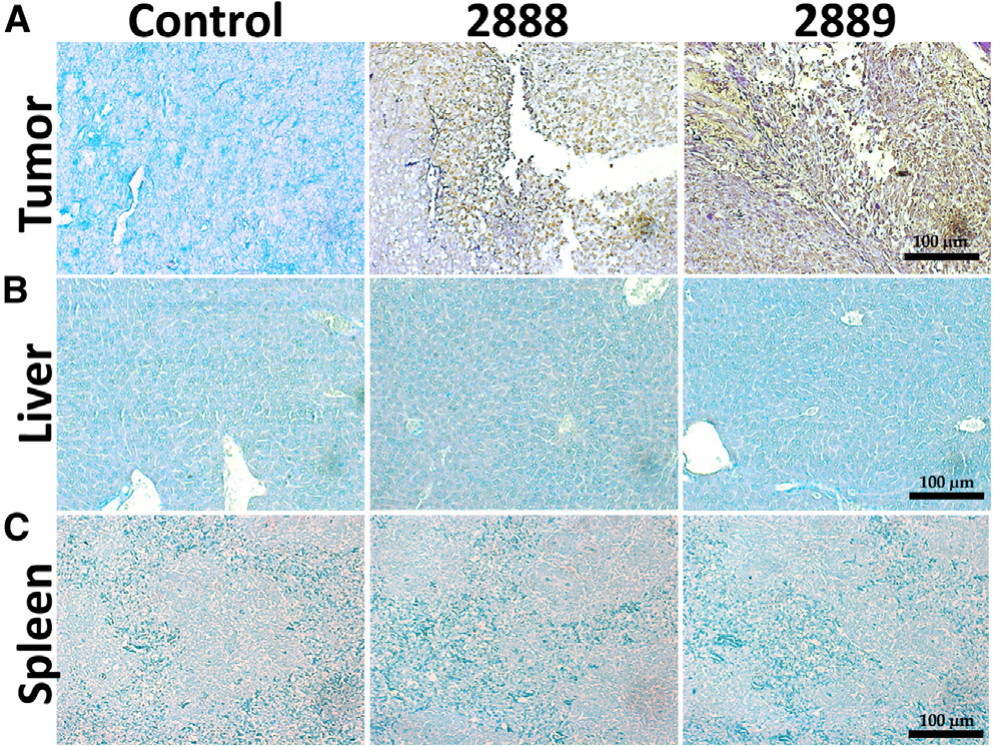
Immunohistochemistry detection of *Salmonella* (A) Tumors, (B) livers, and (C) spleens. The development of a brown color indicates the presence of *Salmonella*, which was detected only in tumor tissue samples, not in the healthy organs.

The histological alterations in the tumors were examined by hematoxylin and eosin (H&E) staining. In both *Salmonella* treated groups, the tumors contained a considerable number of necrotic foci, whereas the cancer tissue architecture was intact in the untreated group (Figure 7A). Both JOL2888 (asRCAS1-delivering) and JOL2889 (vector control) *Salmonella* treatments exhibited immune cell infiltration, confirmed by non-quantitative microscopic observations (Figure 7B). The H&E staining analysis of the *Salmonella*-treated lungs showed a clear, healthy tissue topology, whereas the untreated lungs had multiple micro-metastatic foci (Figure 7C). Further, the localization of *Salmonella* was visualized in the tumors, livers, and spleens by immunohistochemical (IHC) analysis. The brown regions in Figure 8 indicate the presence of *Salmonella*, detected by a *Salmonella*-specific rabbit antibody. Moreover, clear necrotic foci are visible in the brown *Salmonella*-localized regions of the treated tumors. In contrast, the livers and spleens of the treated mice did not show any brown *Salmonella* spots, which substantiates the tumor specificity of the auxotrophic *Salmonella* (Figure 8).

## Discussion

In this work, we aimed to inhibit RCAS1 expression, as it is highly associated with approximately 15 types of cancer.^6^ RCAS1 influences the size and stage of tumors, the degree of invasion, lymphovascular space invasion, and metastasis. RCAS1 not only helps tumor cells to circumvent immune detection, but also promotes cancer stromal remodeling and magnifies the aggressive traits of cancer.^6^^,^^30^ Therefore, inhibition of RCAS1 is envisaged as a broad-spectrum therapeutic strategy. Previous reports suggested that auxotrophic *Salmonella* has excellent tumor-targeting ability^31^ because cancer cells acquire nutrients from their surroundings using various scavenging pathways and altered metabolism from normal tissues.^32^ Tryptophan metabolism is one of the critical factors for cancer progression, because tryptophan derivatives from cancer cells, such as kynurenine, are deleterious to CD8 T cells and thus facilitate immune evasion.^33^ Therefore, in our previous study,^22^ we developed a tryptophan auxotrophic *Salmonella* (JOL2514P3) that can compete for tryptophan and grow specifically in tumors. The tryptophan concentration is generally 2-fold higher in tumor environments than in normal tissues.^34^ The excessive tryptophan in tumors facilitates the proliferation of tryptophan-auxotrophic *Salmonella*, but healthy tissue does not. Thus, tryptophan auxotrophy confers tumor specificity; at the same time, *Salmonella* is known to be an excellent delivery system.^15^^,^^35^ Therefore, in this study, we designed an asRCAS1 and delivered it using the onco-specific tryptophan auxotrophic *Salmonella* delivery strain JOL2848. The genomic *asd* deletion and its exogenous complementation is employed to impose Darwinian selective pressure on the *Salmonella* to retain the plasmid for assured delivery of the active principle, asRCAS1 RNA.^23^ Furthermore, the plasmid cloned with asRCAS1 comprises the RdRp activity of the SFV, which facilitates the cytoplasmic amplification of the desired RNA. The SFV RdRp used in pJHL204 is composed of SFV NSPs 1–4. The plasmid also consists of flanking conserved sequence elements (CSEs) at MCS. The resulting mRNA or asRNA will have the flanking CSEs that will be recognized by RdRp to confer self-amplification.^23^

Preliminarily, we tested the effect of asRCAS1 on 4T1 cell proliferation *in vitro* using lipofectamine-mediated naked DNA delivery and found only a mild decrease in cell proliferation (Figures 1A and 1C). *Salmonella*-mediated delivery, in contrast, exhibited a substantial inhibition of cell proliferation, possibly because of the additional lethality of *Salmonella* (Figures 1A and 1B). The asRCAS1-delivering strain (JOL2868) exhibited a significantly higher inhibitory potential than the vector control (JOL2867), but the difference was not immense. Similar results were observed in the wound healing assay and annexin V – PI apoptosis FACS analysis (Figures 1D and 1E). In brief, the *in vitro* results imply that RCAS1 suppression mildly slowed but did not drastically affect cell viability or proliferation. These results were in agreement with a previous report, which stated that RCAS1 inhibition did not drastically affect cell propagation, but it impaired the ability to induce apoptosis in T cells *in vitro*.^8^ Based on this interpretation, we moved to *in vivo* experiments. The *Salmonella* strains were subjected to *in vivo* passaging and a safety evaluation as a prelude to the *in vivo* anti-tumor evaluation. The safety of the JOL2868 was ascertained by mortality assessment: all mice remained healthy and showed no disease symptoms until 30 dpi (Figure 2A). To increase the adaptability and specificity of the asRCAS1-delivering active strain (JOL2868) in the tumor niche, *in vivo* tumor-to-tumor passaging was done. The results indicate that the passaged strain (JOL2888) exhibited better tumor colonization for a longer time than the unpassaged strain (JOL2868). It suggests that the tryptophan auxotrophy rendered the strain metabolically deficient in normal organs, so it remained safe but maintained high viability and growth in tumors because of the presence of adequate free nutrients. Overall, the results indicate that JOL2888 (asRCAS1-delivering) was not pathogenic upon intraperitoneal, systemic infection and that it had high tumor specificity (Figures 2A–2C). The high tumor specificity was also confirmed by an IHC analysis using an anti-*Salmonella* rabbit antibody. It detected *Salmonella* colonization only in the tumor, not in normal organs such as the liver and spleen (Figure 8). The results are in line with previous reports of ST VNP20009^36^ and ST A1-R.^37^ In a previous study, Δ*SopB*, Δ*SopD*, Δ*LeuB*, and Δ*ArgD Salmonella* were reported as safe with low virulence and high tumor colonization, and the bacterial load decreased after 10 dpi in vital, healthy organs. Comparatively, the JOL2888 (asRCAS1-delivering) also exhibited low virulence, and the bacterial load in healthy organs drastically decreased even at 5 dpi, which indicates a high level of tumor specificity.^38^ Additionally, CBC and body temperature measurements were performed to ensure the safety of the therapeutic strain. Upon wild-type JOL401 infection, significant hypothermia and elevated levels of reticulocytes and monocytes were observed, which signified systemic bacterial infection, inflammation, and innate immune responses.^39^^,^^40^ The high neutrophil count was observed as a response to intracellular bacterial infection.^41^ The reduced lymphocytes and platelet levels indicate the lymphopenia and thrombocytopenia induced by uncontrolled systemic *Salmonella* infection.^42^ Meanwhile, the therapeutic strain exhibited mild or no significant alterations as an evidence of its non-toxicity and safety (Table S3).

To examine RCAS1 inhibition in the tumor following delivery of asRCAS1 via *Salmonella*, we determined RCAS1 expression by western blotting and found that JOL2888 (asRCAS1-delivering) treatment substantially inhibited the expression of RCAS1 in the tumor (Figure 2D). Meanwhile, the vector control strain JOL2889 colonized the tumor but did not considerably affect RCAS1 expression. In parallel, we analyzed expression at the mRNA level by qPCR, which further confirmed RCAS1 suppression in tumors treated with *Salmonella*-mediated asRCAS1 (Figures 5B, 5C, and 5F).

TAMs are pro-tumor macrophages involved in the secretion of chemokines and cytokines that promote tumor development, angiogenesis, metastasis, and immune evasion; thus, they also act as potential diagnostic and prognostic cancer biomarkers.^43^ The, CD68 TAMs are positively correlated with tumor size, metastasis, and angiogenesis.^44^^,^^45^ By promoting epithelial-mesenchymal transition (EMT) and cancer cell extravasation, CCL2 encourages tumor metastasis.^46^ Moreover, CCL2 reduces cytotoxic T cell populations and inhibits T cell effector activity, both of which contribute to the emergence of an immunosuppressive milieu.^47^^,^^48^ The iNOS TAMs cause pro-tumor inflammation, which lowers T cell activation^48^ and inhibits cancer cells from going into apoptosis.^49^ Moreover, they indirectly assist in angiogenesis.^50^ Therefore, to better understand tumor regression and the influence of JOL2888 on TAMs, we examined the levels of these three key TAMs (CD68, CCL2, and iNOS). JOL2888 therapy significantly decreased the number of these TAMs in the tumors (Figure 3) because of its inherent capacity to endure^51^ and eradicate macrophages.^52^^,^^53^ The TAM observations unambiguously confirm that the reduced tumor progression, metastasis, and immune activation seen with JOL2888 (asRCAS1-delivering) treatment were at least partly caused by the loss of TAMs and their signaling molecules. The current results coincide with earlier reports wherein *Salmonella*-delivered legumain and anti-periostin siRNA decreased TAM populations in tumors.^54^^,^^55^ Our T cell population results are also in accordance with previous reports stating that RCAS1/EBAG9 was found in both membrane-bound and secreted forms in cancer.^56^ RCAS1 is a type II membrane protein that can trigger apoptosis by functioning as a ligand for a putative receptor in peripheral lymphocytes and thereby cause immunosuppression.^2^^,^^57^ The membranous RCAS1 undergoes a process called ectodomain shedding in tumor cells, resulting in its secretion into the serum, thereby affecting the systemic T cell population.^58^ The systemic T cell population is crucial for the generation of anti-cancer immune responses.^59^ The untreated control tumor mice have shown a considerably low level of T cell populations. Because of uncontrolled RCAS1 expression from the progressive tumor, the T cell level is less than in naive mice, as evidence of tumor-induced immune suppression. Whereas in JOL2888-treated (asRCAS1-delivering) mice, decreasing the RCAS1 level in tumors hampered its ability to induce apoptosis in T cells, and consequently, the *Salmonella*-delivered asRCAS1 improved CD4^+^ and CD8^+^ T cell levels. In addition, our FACS analysis of splenocytes stimulated with 4T1 cell lysate as a recall antigen indicated significant T cell immune elicitation against cancer (Figure 4). Elevated CD8^+^ cells indicated direct tumoricidal activity, and improved CD4^+^ level signified the supportive anti-tumor role by improving the cytotoxic T cell population, acting as antigen-presenting cells for cytotoxic cells, and helping in the generation of immune memory cells against cancer antigens.^60^ The improved T cell population along with TAMs reduction by JOL2888 (asRCAS1-delivering) *Salmonella* therapy is correlated with a previous report of *Salmonella* that delivers programmed cell death-1 and periostin siRNAs.^55^

We used gene network analyses to identify genes that are directly or indirectly linked with RCAS1 expression and found strong interconnections among the selected cancer-related genes (Figures 5A–5C). Therefore, the effect of *Salmonella*-based RCAS1 inhibition on cancer-related genes was examined by qPCR analysis. High levels IL-4, IL-10, and IL-6 expression have been linked to the development of several cancer types, improved EMT, and the development of treatment resistance via the triggering of anti-apoptotic pathways and metastasis.^27^^,^^61^^,^^62^^,^^63^^,^^64^^,^^65^ These cytokines stimulate cancer cells and stromal cells through autocrine and paracrine mechanisms.^27^ Inhibition of these pro-oncogenic cytokines by 3- to 12-fold correlated with decreased cell proliferation, tumor progression, and metastasis upon JOL2888 (asRCAS1-delivering) treatment. BCL-2 is a leading anti-apoptotic protein^66^ and a 10-fold decrease in BCL-2 expression emphasized one possible reason for the high cancer cell apoptosis that we observed and further substantiated the reduced tumor volume. VEGF is the signaling protein produced by cancer cells that primarily determines the angiogenesis and lymphangiogenesis of a tumor.^67^ VEGF expression was dramatically suppressed after JOL2888 treatment, which corresponds well with the earlier finding that VEGF suppression was coupled with RCAS1 inhibition.^30^ In addition, significant reduction of SNAP23, VAMP7, and NLRC5 proteins indicates the reduction of cancer-associated protein trafficking, unconventional secretome, and tumor growth.^68^^,^^69^^,^^70^ In a nutshell, *Salmonella*-mediated asRCAS1 delivery resulted in the inhibition of multiple pro-tumor genes (Figures 5D–5F) that promote cancer, deflect apoptosis, enhance angiogenesis, and are involved in the cancer secretome. The suppression of these genes was interpreted to be one of the factors involved in tumor retardation and metastasis arrest.

After four doses of JOL2888 (asRCAS1-delivering) treatment, tumor volume, and cancer-induced splenomegaly were drastically decreased, and lung metastasis was completely arrested (Figures 6 and 7C). An inclusive anti-tumor outcome was attained through the multifactorial therapeutic potential of JOL2888: the inhibition of RCAS1 expression enabled T cell immune elicitation, reduced the TAM population, and suppressed the expression of pro-oncogenic genes alongside the inherent tumor specificity and lethality of JOL2888. In addition to the FACS analysis of T cell immune elicitation, H&E staining also confirmed vast immune cell infiltration in the treated tumors (Figure 7B). Further, H&E staining of the lungs confirmed the complete cessation of metastasis (Figure 7C). The previous report of ST VNP20009 did not show a reduction in metastasis,^71^ but in another study, metastasis inhibition was observed upon treatment with *Salmonella*-delivering siRNA against STAT3,^72^ and that coincides with our present observations. As a live auxotroph, *Salmonella* can self-proliferate specifically in the tumor environment, even in the hypoxic core region, and thus exhibit an inherent lethal effect against tumor cells. In addition to RCAS1 inhibition, and the default anti-tumor potential activity of *Salmonella* by triggering innate immune responses such as TLR-5 activation^73^ and reducing immunosuppressive myeloid-derived suppressor cells and regulatory T cell populations^74^ in tumor also played a major role in the induction of anti-tumor responses. For that reason, the vector control strain (JOL2889) also demonstrated significant tumor reduction. As can be seen from our results, when compared with the active strain delivering asRCAS1 (JOL2888), the JOL2889 strain showed comparatively less but still considerable activity in TAM reduction, T cell elicitation, and immune activation. This is the principal advantage of using *Salmonella* as a delivery system for anticancer therapeutics: it has a default, multifactorial, antagonistic potential against tumor cells. Conversely, other anticancer delivery systems, such as viral vectors and nanoparticles, are devoid of tumor-specific proliferation and inherent lethality.^75^^,^^76^

In summary, we used a tryptophan auxotrophic *Salmonella* strain to deliver an RNA against RCAS1 to tumors. The safety potential was validated *in vivo*, and the tumor-targeting ability of the active strain was improved by *in vivo* passaging. The inflammatory response upon bacterial infection in humans is very critical; therefore, rapid clearance of *Salmonella* from healthy organs becomes a key factor. The high tumor specificity and rapid clearance from healthy organs exemplified the suitability of JOL2888 in future human applications. The tumoral delivery of asRCAS1 and consequent RCAS1 inhibition was demonstrated. Based on these findings, we conjecture that the decrease in TAM level, elicitation of a T cell immune response, and suppression of pro-tumor genes by *Salmonella*-mediated asRCAS1 delivery resulted in the drastic tumor reduction and prevention of metastasis. The RCAS1, an underrated cancer antigen, became a major, frequently detected antigen in human cancers; thus, the JOL2888 gained greater applicability in human cancers. Being a facultative anaerobe, this *Salmonella* can penetrate the tumor core region and inhibit the hypoxic cancer cells, which is a determining factor of tumor re-emergence. Thus, it may decrease the chances of tumor re-emergence, which is a possible advantage over conventional approaches. This study provides new insights for applying engineered *Salmonella* as a safe tumor-targeted delivery system with intrinsic anti-tumor therapeutic potential. The results of this investigation exemplify JOL2888 as a potential option for combinatorial oncotherapy with chemo- or radiotherapy. Moreover, it could be a reliable alternative for treating chemo/radiation-resistant cancers.

## Materials and methods

### Bacterial strains, cell line, plasmids, and primers

The bacterial strains, plasmids, and primers are tabulated in Table S1. The bacterial cultures were grown in LB broth (BD) and maintained in LB agar with appropriate antibiotics. The 4T1 mouse mammary carcinoma cell line was grown in RPMI cell culture medium supplemented with 10% FBS (Gibco) and a 1% 100× penicillin and streptomycin antibiotic solution (P/S) at 37°C in 5% CO_2_.

### Ethics statement

The *in vivo* experiments were carried out using 6-week-old female BALB/c mice procured from Koatech. All the mice experiments were designed by considering replacement, reduction, and refinement as per the guidelines.^77^ All mice were maintained as per standard protocol and fed with a standard regimen at the Animal Housing Facility of the College of Veterinary Medicine, Jeonbuk National University. All *in vivo* experiments involving experimental animals were approved by the Jeonbuk National University Animal Ethics Committee (JBNU 2021-027) under the Korean Council on Animal Care and the Korean Animal Protection Law, 2001, article 13.

### Construction of the therapeutic strain

In this study, we designed an lnc asRNA of 312 bp against RCAS1 mRNA. The asRNA sequence was selected using the criteria of high specificity and least self-complementarity, which was checked using an online server (http://biotools.nubic.northwestern.edu/OligoCalc.html). The asRNA secondary structure, base pair probability, and positional entropy were analyzed on an online server (http://rna.tbi.univie.ac.at/cgi-bin/RNAWebSuite/RNAfold.cgi) offered by the Institute for Theoretical Chemistry, University of Vienna, Austria. The DNA sequences encoding the asRNA against RCAS1 were custom synthesized by Cosmo Genetech and cloned into the pJHL204 expression plasmid, at downstream of the SV40 promoter using ApaI and PacI restriction sites at the 5′ and 3′ ends, respectively. The cloned plasmid delivers the desired DNA/RNA to eukaryotic cells via *Salmonella* infection. The cloned plasmid comprising the asRCAS1 was transformed into *Salmonella* (JOL2848 - Δ*trpA ΔtrpE Δasd*), and the positive strain was designated as JOL2868. The genotypic auxotrophic deficiency of *asd* was complemented by the pJHL204 plasmid, which acts as a non-antibiotic selection marker to retain the plasmid in *Salmonella* (Figure S1).

### Cell proliferation and wound healing assay

The 4T1 cell culture at 40% confluency in 24-well plates were subjected to Lipofectamine 3000 transfection using 500 ng/well purified, asRCAS1 cloned plasmid and null vector, as per the manufacturer’s instructions. Similarly, *Salmonella* JOL2868 and JOL2867 were used for bactofection at a multiplicity of infection (MOI) of 50 (1.25 × 10^7^ CFU) for 3 h. After infection, the cells were washed with RPMI medium, and extracellular bacteria were killed using 100 ppm of gentamycin for 2 h. After transfection/bactofection, the cells were washed with RPMI and incubated for 3 days in RPMI supplemented with 10% FBS + 1× P/S. Then, the MTT colorimetric assay and microscopic observation were done using standard protocols.

Similarly, the effect of *Salmonella* JOL2868 on cell proliferation was tested with a wound-healing assay. The 4T1 cell monolayers were made in a 12-well plate, and a linear scratch was made on them using a 200-μL tip. Then, the cell monolayer was washed, and bactofection was done as described earlier. After bactofection, the cells were incubated for 3 days, and then the wound healing in the cell layer was microscopically observed.^78^

### Flow cytometric analysis of apoptosis

The 4T1 cell cultures at 90% confluency in a 24-well plate were subjected to bactofection using JOL2868 and JOL2867 at 20 MOI (5 × 10^6^ CFU). The bactofection was done by infecting the cells for 3 h, followed by gentamycin (100 ppm) treatment for 2 h. Then, the cells were washed thrice with RPMI media and incubated with 10% FBS supplemented RPMI for 24 h. After incubation, the cells were treated with Trypsin-EDTA (0.25%) for 3 min. After cell detachment, cell suspensions were centrifuged at 400×*g*, washed with PBS, and suspended in RPMI media. Subsequently, cells were stained and processed for a FACS analysis using an Annexin V apoptosis detection kit I (BD Pharmingen), according to the manufacturer’s instructions.^79^

### Survival assay for safety assessment

Mice (n = 6/group) were intraperitoneally infected once with JOL2868 or JOL401 (wild type) at a CFU of 1 × 10^7^ in 100 μL per mouse. The mice were monitored for disease symptoms every day, the number of dead animals was recorded, and a survival graph was plotted.

### Tumor induction and *in vivo* passaging

The 4T1 cell suspensions of 1 × 10^5^ cells in 100 μL were subcutaneously injected into the second mammary fat pads of mice and allowed to develop into tumors for 15 days. The tumor-bearing mice (n = 2) were then intraperitoneally infected once with the developed onco-therapeutic *Salmonella* candidate (JOL2868) at 1 × 10^6^ CFU/100 μL per mouse. At 3 dpi, the mice were sacrificed, and the tumors were collected for *Salmonella* isolation. The tumor homogenate was subjected to serial dilution and spread plating on brilliant green agar (BGA) plates, and *Salmonella* was isolated. The isolate was confirmed for asRCAS1 by PCR; after confirmation, the passaged strain was used for subsequent infection. In that way, three passages were done.^80^

### Surface body temperature and CBC analysis

To further confirm the safety potential of the therapeutic *Salmonella* strain, the surface body temperature was measured at ano-genital area of mice using a non-contact infrared thermometer (Apollo non-contact infrared thermometer, Sangon Co., Ltd.) after 3 days of *Salmonella* infection through the intraperitoneal route. The measurements were taken with a readout time of 3–4 s and the thermometer was kept at a distance of 1–2 cm from the mice.^81^

Subsequently, the CBC was determined in blood samples (n = 8) from ST2888 (*in vivo* passaged therapeutic strain) and JOL401 (virulent wild type) *Salmonella*. PBS inoculated mice were subjected as control. After 3 days of infection, mice were sacrificed, and blood samples were collected directly from heart and mixed with 20 μL 10% EDTA solution. A total of 200 μL of each blood sample was subjected to CBC measurement using the ProCyte Dx Hematology Analyzer (IDEXX).^82^

### Determination of the tumor specificity of the therapeutic *Salmonella* strain

Two groups of tumor bearing mice (n = 8) were intraperitoneally infected once with passaged (JOL2888) and non-passaged (JOL2868) therapeutic *Salmonella* candidate strains at a CFU of 1 × 10^6^ in 100 μL PBS. The mice were sacrificed at 3, 5, 7, and 14 dpi, and tumor, liver, and spleen samples were collected. A CFU assay of the tissue homogenate was performed by serial dilution and spread plating on BGA plates.

### *In vivo* assessment of therapeutic potential

Three groups of mice (n = 8) were taken, and tumor induction was done as described earlier. The group 1 tumor-bearing mice were intraperitoneally infected with JOL2888 at a CFU of 1 × 10^6^ in 100 μL PBS. The bacterial treatment was done four times at 7-day intervals. Similarly, the vector control strain JOL2889 was used to treat the group 2 mice, and the untreated control group 3 received sterile PBS. All mice were monitored daily for disease symptoms, and tumor size was measured with a Vernier caliper every 5 days. Two weeks after the last treatment, all the mice were sacrificed, and their lungs, spleens, and tumors were collected. The maximum permitted tumor size was 2 cm^3^ was ensured as per the latest Institutional Animal Care & Use Committee policies, “Standard on Tumor Production and Cancer Research in Mice and Rats.” The tumor volume was measured using the formula: tumor volume = (length × width × height)/2. The lung samples were subjected to gross examination for metastatic nodules.^83^

### Determination of RCAS1 expression

Tumor samples were homogenized and sonicated for protein isolation. After sonication, the suspension was centrifuged at 12,000 rpm for 10 min at 4°C, and the supernatant was stored at −80°C. The protein samples were subjected to SDS-PAGE analysis followed by western blotting. The blotted membrane was blocked with a 5% skim milk suspension and then incubated at 4°C overnight with a 1:500 ratio of poly-clonal mouse RCAS1 primary antibody. After washing, rabbit anti-mouse IgG-HRP secondary antibody (Southern Biotech) at a ratio of 1:6,000 was added and incubated for 1 h at room temperature. After incubation, the membrane was washed and developed using a WESTSAVE gold chemiluminescence kit (Abfrontier). In parallel, GADPH was tested as a housekeeping control using GADPH mouse monoclonal antibody (ABclonal) at a 1:5,000 dilution.

### RCAS1 and the cancer-associated gene network qPCR analysis

To identify the genes connected with RCAS1 and understand the links and inter-dependency among them, we conducted gene network analyses through the STRING database (https://string-db.org/), GeneMANIA (https://genemania.org/), and KEGG (http://www.genome.jp/kegg).^28^ Based on these results, we selected cancer-associated genes and conducted a qPCR analysis to study the effects of RCAS1 suppression (via the lnc asRCAS1) in the tumor. The primers used for this analysis are listed in Table S2.

### Assessment of T lymphocytes and TAMs by FACS analysis

Splenocytes were isolated from the control and treated mice using a standard protocol.^84^ The isolated splenocytes were induced with 500 ng/mL 4T1 cell lysate as a recall antigen for 72 h. After incubation, the splenocytes were washed and stained with DAPI, FITC-labeled anti-CD8a, PerCPVio700-labeled anti-CD4, and PE-labeled anti-CD3e FACS antibodies (Miltenyi Biotec) in the same panel. After 40 min of staining, cells were washed twice with FACS running buffer and subjected to FACS analysis. The CD3, CD4, and CD8 sub-populations were analyzed using a MACSQuant flow cytometer (Miltenyi Biotec). Live cells were gated as DAPI negative, then CD3-positive cells were gated for CD4 and CD8 sub-population analysis.

Similarly, TAMs were isolated using Ficoll-Paque plus (Cytiva) after the tumor tissue was digested using 0.05% collagenase IV, as described by Li et al.^85^ Briefly, live, vasculated tumor tissues were taken and chopped into 1- to 2-mm^3^ pieces for easier digestion. Then, 2 mL 0.05% collagenase IV in sterile PBS was added and incubated for 3 h in a CO_2_ incubator. Every 1 h the tissue suspensions were pipette mixed; after incubation, the suspensions were filtered through a 100-μm mesh screen. The filtered cell suspensions were washed with RPMI media and stained with fluorescently labeled FACS antibody markers in the same panel. To gate the live cells, DAPI (-ve) was used; CD45 (PerCP), CD11b (PE-Vio 770), were used as lymphocyte and monocyte markers respectively. The F4/80 (PerCP-Vio 700), marker was used as specific marker for mature macrophage and iNOS (FITC), CD68 (PE), and CCL2 (APC) were used as TAMs markers. The DAPI, CD45, CD11b, F4/80, and CD68 were stained by surface staining procedure and subsequently, the CCL2 and iNOS intracellular staining was done after Brefeldin A and membrane permeabilization buffer treatment for intracellular fixation (Thermo Scientific). To avoid non-specific binding, anti-mouse Fc block (BD Pharmingen TM 553141) was used before surface staining as per manufacturer’s instructions. The FACS markers were procured from Miltenyi Biotec. The TAMs gating strategy is illustrated in Figure S2.

### Histopathological and IHC analyses

The tumor and organ samples collected after treatment were processed for H&E staining using a standard protocol.^86^ Live, vasculated tumor tissues were taken for tissue processing and the immune cell infiltration was identified by clusters or aggregates of individual cells with a high nuclear-to-cytoplasmic ratio, which are mostly immune cells. In parallel, tumor samples were processed for IHC analysis, to detect the presence of *Salmonella* using anti-*Salmonella* rabbit primary antibody (1:200 dilution) and goat anti-rabbit IgG-HRP secondary antibody (1:250 dilution) to ensure the tumor specificity of the therapeutic strain.^87^ After incubation with the 2° antibody, a 3,3′diaminobenzidine substrate was used for color development. Randomly, two specimens from each tumor were subjected, and all the regions of the tumor specimens were scanned. Six fields per specimen were captured. Representative images are shown in the results figure.

### Statistics

GraphPad Prism 9.0 software (GraphPad) was used to analyze the data via Student’s t-test and ANOVA. A p value of less than 0.05 was regarded as significant. Data are displayed as the mean ± SEM in graphs with ∗p < 0.05, ∗∗p < 0.01, and ∗∗∗p < 0.001.

## Data and code availability

The datasets obtained and/or analyzed during the current study are available to reproduce these findings from the corresponding author upon request.

## Supplemental information

Document S1. Figures S1, S2, and Tables S1–S3

Document S2. Article plus supplemental information
