# Supplementary material for: Tumor-targeted delivery of lnc antisense RNA against RCAS1 by live-attenuated tryptophan-auxotrophic Salmonella inhibited 4T1 breast tumors and metastasis in mice
Source: Mol Ther Nucleic Acids. 2023 Oct 13;34:102053. doi: 10.1016/j.omtn.2023.102053 (PMC10628790; doi:10.1016/j.omtn.2023.102053)
Supplement: Document S1. Figures S1, S2, and Tables S1–S3 [file mmc1.pdf]

OMTN, Volume *34*

Supplemental information

Tumor-targeted delivery of lnc antisense RNA against RCAS1 by live-attenuated tryptophan-auxotrophic *Salmonella* inhibited 4T1 breast tumors and metastasis in mice

Chandran Sivasankar, Chamith Hewawaduge, Pandiyan Muthuramalingam, and John Hwa Lee

Table S1. List of bacterial strains and plasmids used in the study

Bacteria/Plasmid	Genotypic characteristics	Reference
***S.* Typhimurium**		
JOL401	*Salmonella* Typhimurium wild type	[16]
JOL2514	JOL401 Δ*trpA* Δ*trpE*	[21]
JOL2848	JOL2514 Δ*asd*	This study
JOL2868	JOL2848 + pJHL204 + lnc-asRCAS1	This study
JOL2888	JOL2868 thrice *in vivo* passaged	This study
JOL2867	JOL2848 + pJHL204 (vector control)	This study
JOL2889	JOL2867 thrice *in vivo* passaged	This study
E. coli		
DH5α	*E. coli* F^{-}Φ80dlacZΔM15Δ (lacZYA-argF) U169recA1 endA1 hsdR17(rk-, mk^{+}) phoA supE44 thi1 gyr A96 relA1λ-	Lab stock
E. coli 232	F – λ – φ80 Δ(lacZYA-argF) endA1 recA1 hadR17 deoR thi-1 glnV44 gyrA96 relA1 ΔasdA4	Lab stock
BL21(DE3)	F^{-},*omp*T, *hsd*S_B (r^{-}_{B}, m^{-}_{B}), *dcm, gal,* λ (DE3)	Lab stock
JOL 2981	DE3 carrying pET28(a) + RCAS1	This study
Plasmids		
pET28a(+)	IPTG-inducible expression vector; Kanamycin resistance	Novagen, USA
pJHL204	SFV replicon, asd+, CMV promoter, SV40 promoter, pBR322 ori	Lab stock
pKD3	oriR6Kgamma, bla (amp^R), rgnB (Ter), cat^R, FRT	
pKD46	oriR101-repA101ts; encodes lambda red genes (exo, bet, gam); native terminator (tL3); arabinose-inducible promoter for expression (ParaB); bla	
pCP20	encodes FLP, $ampicillin^R$ and Cm^R	

Table S2. List of primers used in the study

Primers	Genotypic characteristics	Reference
**Construct primers**		
asd pKD3	Forward – TGA AGG ATG CGC CAC AGG ATA CTG GCG CGC ATA CAC AGC ACA TCT CTT TGG TGT AGG CTG GAG CTG CTTC<br>Reverse – TAT CCG GCC TAC AGA ACC ACA CGC AGG CCC GAT AAG CGC TGC AAT AGC CAA TGG GAA TTA GCC ATG GTCC	[16]
asd - inner	Forward – CAT GGT AGA GGA GCG CGA TT<br>Reverse – TAC CGC CCA CAA AGG TCT TC	[16]
asd - outer	Forward - GCG ACG GAA ATG ATT CCC TT<br>Reverse - AAG CTA CCC TTA AAG AAT AGCC	[16]
asRCAS1	Forward – GGG CCC TAC CGG TAG TGA<br>Reverse – TTA ATT AAA CCA CAG TAC AGG AAC TTC	This study
RCAS1 RT PCR	Forward – TAT TTA AAG TTT GTA CCT GC<br>Reverse – TAG CAG AGT AG TTT CCC	This study
Optimized RCAS1	Forward – GAA TTC ATG GCT ATT ACC CAA<br>Reverse – GTC GAC TTA CGA CAA TTT CAC	This study
**qPCR cytokine primers**		
RCAS1	forward- GGA ACA ACT GGA ACC TGA CTA C<br>reverse- AAA AAC CCG TGC TAC CAT CTG	[21]
IL-4	Forward – ACA GGA GAA GGG ACG CCA T<br>Reverse – GAA GCC CTA CAG ACG AGC TCA	[21]
IL-10	Forward – GGT TGC CAA GCC TTA TCG GA<br>Reverse – ACC TGC TCC ACT GCC TTG CT	[21]
IL-6	forward- GAG GAT ACC ACT CCC AAC AGA CC<br>reverse- AAG TGC ATC ATC GTT GTT CAT ACA	[21]
INFγ	Forward – TCA AGT GGC ATA GAT GTG GAA GAA<br>Reverse – TGG CTC TGC AGG ATT TTC ATG	[21]
TNFα	Forward – CAT CTT CTC AAA ATT CGA GTG ACA A<br>Reverse – TGG GAG TAG ACA AGG TAC AAC CC	[21]
IL1β	Forward- TTC ACC ATG GAA TCC GTG TC<br>Reverse- GTC TTG GCC GAG GAC TAA GG	[21]
BCL-2	Forward- CCT GTG GAT GAC TGA GTA CCT G<br>Reverse- AGC CAG GAG AAA TCA AAC AGA GG	This study
Bax	Forward- AGG ATG CGT CCA CCA AGA AGC T<br>Reverse- TCC GTG TCC ACG TCA GCA ATC A	This study
BCL-XL	Forward- GCC ACC TAT CTG AAT GAC CAC C<br>Reverse- AGG AAC CAG CGG TTG AAG CGC	This study
VEGF	Forward- GCG AAG CTA CTG CCG TCC<br>Reverse- TCT GCA TGG TGA TGT TGC TC	This study
p21	forward- CGA GAA CGG TGG AAC TTT GAC<br>reverse- CAG GGC TCA GGT AGA CCT TG	[21]
p27	forward- TCA AAC GTG AGA GTG TCT AAC GG<br>reverse- AGG GGC TTA TGA TTC TGA AAG TCG	[21]
p53	forward- GTC ACA GCA CAT GAC GGA GG<br>reverse- TCT TCC AGA TGC TCG GGA TAC	[21]
SNAP 23	Forward- TCC ATA CAT CAC CGT CTC TTC<br>Reverse- CCA CAA AAC CAA AAC CAA ACC	This study
Syntaxin 4	Forward- AAAAGCCATAGAGCCCCAG<br>Reverse- TCG TCA GAC ACC ATT CCA G	This study
VAMP7	Forward- TTC TTT TTG CTG TTG TTG CC<br>Reverse- TCT TGA ACC GTA AGT TGT CTG	This study
C erb B	Forward- CCC TAC AAC TAC CTC TCC AC<br>Reverse- TTC TTG CAG CCA GCA AAC	This study
SALL1	Forward- CCC CAT CCC TAT TAG CCA TTC<br>Reverse- TAC TCT CTT CAC CCT TGC C	This study
NLRC5	Forward- GGA AGA ACA GCA AGA AGC AG<br>Reverse- TGT GGG GAG TGA GGA GTA AG	This study
GADPH	Forward – TCA CCA CCA TGG AGA AGG C<br>Reverse – GCT AAG CAG TTG GTG GTGC A	

Table S3: Body temperature and complete blood count analysis of ST2888 safety assessment. The experiment was performed twice with four biological replicates (n=4). The data were analysed by ANOVA using Tukey's post hoc test. *p < 0.05; **p < 0.01; ***p < 0.001; ****p < 0.0001.

		PBS		ST2888		ST401	
	Units	Avg	SD	Avg	SD	Avg	SD
Body Surface temperature	°C	32.1	0.33	32	0.48	30.50*	0.39
RBC	10^6/µL	9.1	0.04	8.96	0.27	9.41	0.39
HCT	%	43.15	0.06	41.9	3.35	45.85	2.37
HGB	g/dL	14.55	0.06	14.55	0.4	14.7	0.69
MCV	fL	45.8	0.46	44.55	0.17	44.35	0.29
MCH	pg	15	1.15	16.25	0.06	15.75	0.17
MCHC	g/dL	34.95	0.4	36.45	0.06	35.6	0.12
RDW	%	23.85	0.17	25.65	0.98	23.9	1.04
%RETIC	%	3.2	0.46	3.1	0.58	5.75*	0.4
RETIC	10^3/µL	293.9	42.26	364.85*	41.4	626.75**	50.86
WBC	10^3/µL	6.84	0.25	7.02	1.11	6.66	0.09
%NEU	%	14.1	0.12	18.2	3.7	41.15**	0.4
%LYM	%	83	0.23	79.1	2.19	48.95*	1.67
%MONO	%	1.2	0.35	1.35	0.75	8.05****	1.21
%EOS	%	1.75	0.06	1.35	0.75	1.5	0.12
%BASO	%	0	0	0	0	0.35	0.17
NEU	10^3/µL	1.04	0.11	0.48	0.19	2.33**	0.06
LYM	10^3/µL	4.98	0.79	6.09	0.67	2.77*	0.05
MONO	10^3/µL	0.09	0.03	0.07	0.03	0.46**	0.08
EOS	10^3/µL	0.13	0.02	0.03	0.01	0.09	0.01
BASO	10^3/µL	0	0	0	0	0.02	0.01
PLT	10^3/µL	714.5	15.59	590*	80.83	216.50***	24.83
MPV	fL	8.6	0.23	9.45	0.75	9.95	0.29
PCT	%	0.69	0.06	0.57	0.12	0.22	0.03

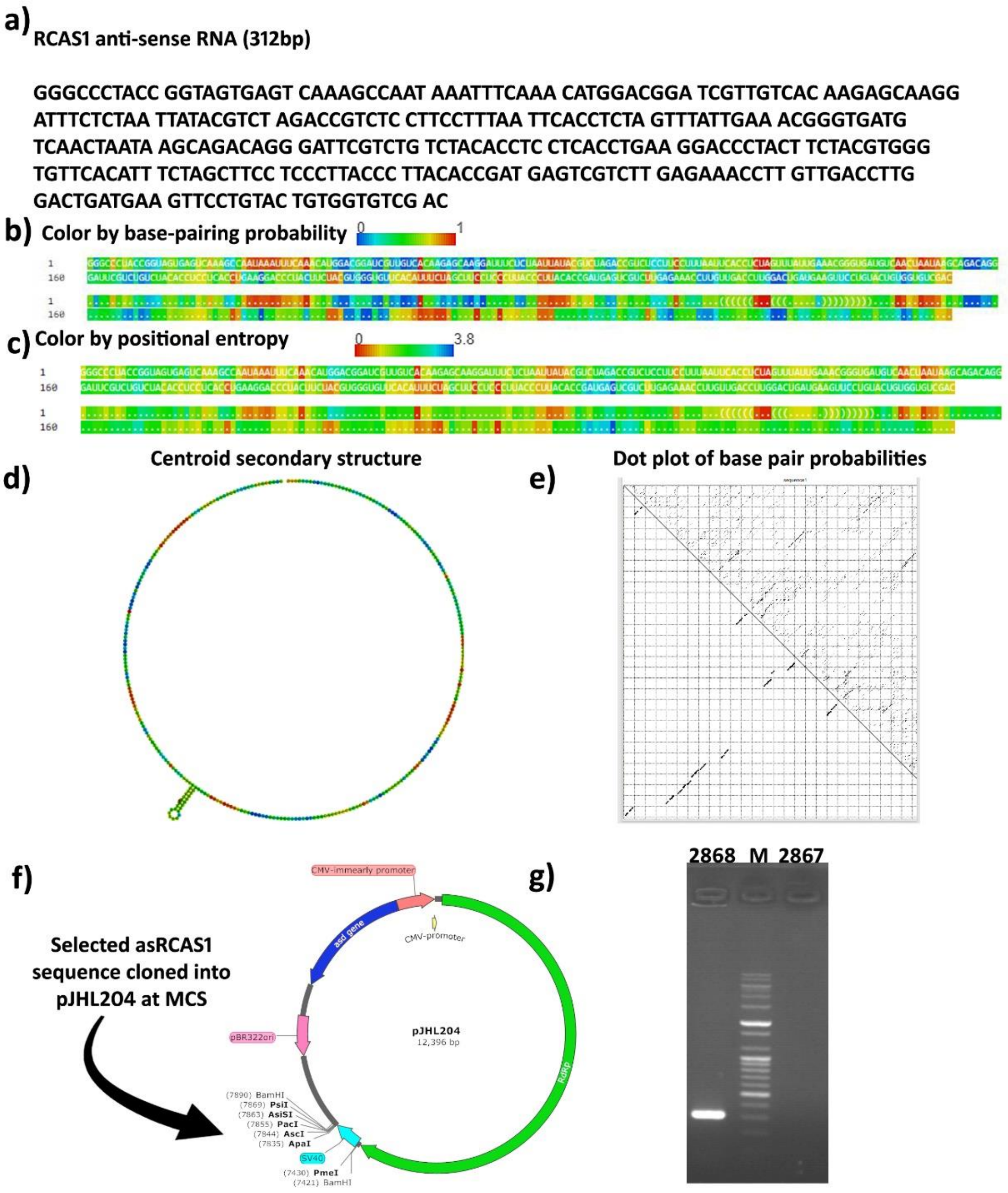

Figure S1. Anti-sense RNA against RCAS1. a) DNA sequence of RCAS1 anti-sense RNA. b) RNA base-pairing probability. c) positional entropy of the RNA sequence. d) centroid secondary structure prediction. e) dot matrix of RNA base-pairing probability. f) pJHL204 map and cloning strategy of asRCAS1. The sequence was cloned with the orientation of ApaI and PacI sites at 3' and 5' respectively, at MCS under the SV40 promoter of pJHL204 plasmid. g) asRCAS1 positive strain (ST2868) at left to marker DNA with a band near 300bp, and asRCAS1 negative vector control strain (ST2867).

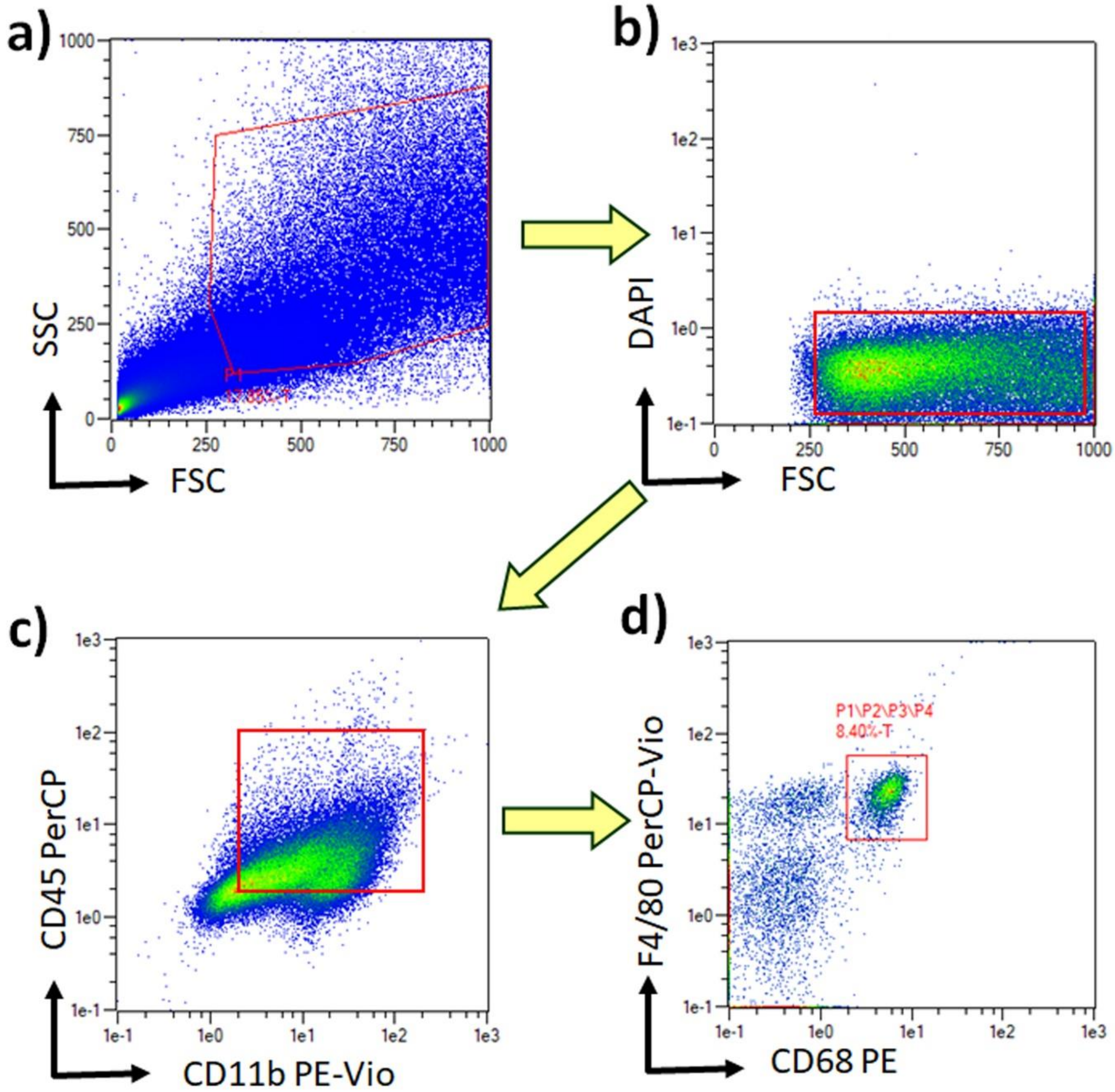

Figure S2. Gating strategy of the TAMs. a) The forward scatter (FSC) and side scatter (SSC) dot plot of whole tumor cell suspension and possible region of macrophage was selected. b) live cells were gated as DAPI negative. c) then lymphocytes and macrophages were gated as CD45 and CD11b double positive cells. d) subsequently mature macrophages and selected TAMs were gated as F4/80 and CD68/ iNOS/ CCL2 double positive cells.
